# Do Sports Clubs Contribute to the Accumulation of Regional Social Capital?

**DOI:** 10.3390/ijerph17145257

**Published:** 2020-07-21

**Authors:** Elżbieta Biernat, Hanna Nałęcz, Łukasz Skrok, Dawid Majcherek

**Affiliations:** 1 Department of Tourism, Collegium of World Economy, SGH Warsaw School of Economics, al. Niepodległości 162, 02-554 Warsaw, Poland; 2 Department of Child and Adolescent Health, Institute of Mother and Child, Kasprzaka Str. 17A, 01-211 Warsaw, Poland; hanna.nalecz@imid.med.pl; 3 Department of Business Economics, Collegium of World Economy, SGH Warsaw School of Economics, al. Niepodległości 162, 02-554 Warsaw, Poland; lskrok@sgh.waw.pl; 4 Collegium of World Economy, SGH Warsaw School of Economics, Aleja Niepodległości 162, 02-554 Warsaw, Poland; dm53757@doktorant.sgh.waw.pl

**Keywords:** sports activity, sports club, social capital, regional level, public health

## Abstract

Social capital (SC) affects quality of life, sport behaviours and health in individual and community context. The aim was to analyse how sports activity (SA) contributes to SC accumulation in a post-transformation country. A combination of four longitudinal, nationwide datasets was used. Instrumental variable method was applied. Results show that in 19+ Poles, on a regional level, SA improves SC. This suggest that sports clubs and at least some types of sports infrastructure can constitute a valid tool for social policies aiming at improving social involvement. The self-triggering character of SA is a valuable asset for social impact regionally and beyond. It is important to estimate the changes in a long-term perspective, due to the inertness of the SC.

## 1. Introduction

The concept of social capital (SC) was popularised in the 1980s by Coleman [1] and Putnam [2], and later by Bourdieu [3] and Fukuyama [4]. Since then, many SC concepts have been developed. It has been defined according to: the field of science (e.g., sociology, economy, political science and geography), social range (micro—individuals, meso—local communities, macro—regions or countries), the SC type according to the different kind of bonds that connect people (bonding, bridging and linking social capital). Measuring SC and determining what it consists of is not easy. Apart from the variety of definitions resulting in the lack of a uniform methodology [5], SC includes different dimensions. Among them, Claridge [6] lists: trust, norms and principles defining social activities, types of social interaction and dimensions of social networks, group activities, volunteerism, daily interactions with other people, neighbourly relations and sense of community. Pawlowski and Schüttoff discuss the relation between sports and SC using four-dimensional framework: personal relationships, social network support, civic engagement and trust and cooperative norms, intersected with two types of SC accumulation—vertical (reflecting bridging SC) and horizontal (reflecting bonding SC) [7]. It is problematic to directly measure indicators important for SC such as trust or truthfulness. However, this can be done indirectly, by looking at the factors that build these indicators, expressing their existence (SC measures) [8]. Initially, SC was measured by means of participation in voluntary groups or of their number, voter turnout, magazine readership and the level of altruism through the share of charity spending and the number of blood donors [9]. Then, other measures were added, e.g., the percentage of people who did not remove their number from the directory service or wrote off donations in their tax return, as well as the number of bars, cafés and sports associations [10]. Measure of sports also differ. For example, Downward, Hallmann and Rasciute [11] analysed the impact on SC of sporting activity undertaken in the last four weeks, while Delaney and Keaney [12] examined the numbers of people who participate in sport, and the ways of participation: how many volunteer, how many are members of sports clubs and how many attend sporting fixtures. Groups related to sports are increasingly taken into account as a factor linked to building SC [13].

Researchers demonstrate the coexistence of high levels of sports activity (SA) and SC and the relationship of SA with individual dimensions of SC using varied datasets and methodologies. In particular, while looking at the research carried out on an individual level, according to Ball et al. [14], SA is positively correlated with mutual neighbourly trust and social activity (the authors used a cross-sectional dataset from Melbourne and multivariate regression models). Harvey et al. [15], by conducting a survey in Ontario and Quebec, show that volunteerism in sports positively correlates with various measures of a person’s social network. Brown [16], by conducting correlation analysis using results from a survey of community organisations members in Lund (Sweden) and Ballarat (Australia), shows that members of sport or recreation organisations, in comparison with other organisations, had higher trust in government, greater tolerance and better relations with neighbours. In turn, at both individual and aggregated, national level, using multivariate regression analysis, Delaney and Keaney [12] show that membership of sports clubs correlates positively with sociability and trust in institutions. Schüttoff et al. [17], using matching estimation and panel data on adolescents in Germany, claim that regular SA fosters a greater willingness to help friends/neighbours and to belong to civic organisations, political parties and local governments, and that exercising in sports clubs means a greater willingness to engage in voluntary activities. Felfe et al. [18], using two separate datasets—a cross-sectional and longitudinal one—on German children and matching estimation argue that sport club participation reduces peer problems. Pawlowski et al. [19], using longitudinal data on children in Peru and matching estimation, report that participation in sports group leads to feeling friends’ support. Downward et al. [20], using multinational, individual, cross-sectional dataset and instrumental variables (IV) estimation, claim that while generalised trust is positively correlated to club membership, the causal relation from the latter to the former is not confirmed. Skrok et al. [21] using individual, longitudinal data on adults in Poland and matching estimation, argue that, while no indication of impact on generalised trust can be identified, sports activity can positively influence size and depth of social networks, as well as propensity to engage in pro-social activities and associational behaviour. Finally, Di Bartolomeo and Pappa show, using an experimental design, that even short involvement in exercise can lead to decisions reflecting greater trust and prosocial behaviour [22].

As shown above, empirical literature suggests that SA, through its positive impact on various SC dimensions, can strengthen it. One can observe, however, that researchers frame this issue in many different ways—among others, that sport is a social activity, that it plays a valuable role in building a common identity, that membership in sports clubs and teams is one of the key forms of associational life and finally, that sports groups form networks that go beyond the participants themselves [12]. In particular, the creation of community associations is one of the SC dimensions. In this context, it is important to note that Brown shows that membership of sports and recreation organisations leads to exceptionally high scores in some SC measures [16]. Such SC presence in the structures of sports participation seems to be of key importance for the existence and continuation of these communities [23] and for members to remain in an organisation and continue their activity, including physical activity [15]. It can also, as Skrok et al. [21] or Eime et al. [24] say, provoke attitudes and behaviours that will favour SC. For example, according to Harvey et al. [15] staying in active sports communities can encourage their members to remain physically active and reap the psychological benefits of membership. Furthermore, using microeconomic theory and individual-level data for the UK, Downward and Riordan [25] claim that participation in sport strongly promotes its continuation “for example, the consumption capital developed from the acquisition of skills and capabilities to engage in sport.” This result is also shown empirically for Poland [21]. Therefore, scientific research results cited above may suggest the existence of the strong self-effect of SA.

In this context, investigating the relationship between sport participation and SC, as well as the self-triggering nature of the former—especially in countries such as Poland, where both SC and SA are low—is important for understanding these phenomena and for undertaking corrective actions.

An analysis of existing literature on the relationship between SA and SC indicates three important issues. The first is that SC tends to favour SA [25], which results in positive correlation between them. However, as mentioned before, there are several reports, although less frequent, which show the opposite relationship. As shown, however, most of them concern studies on children [18,19] or adolescents [17], only two studies conducted causal analysis on a dataset on adults [20,21]. The second issue is that it is not shown that SA leads to improvement in all measures, including generalized trust [20,21] but rather a tendency to establish relationships with other people [17,19,20,21] or engage in association behaviour or pro-social activities [17,21]. The third issue is that, so far, the majority of the research has been conducted in Western European and North American countries or developing countries. This is a significantly different context of research from that of post-transformation countries, such as Poland, both in terms of SA (especially the availability of infrastructure) and SC. Limited publications concerning Poland have been published in non-English-speaking journals in recent years [26,27] and only a few works reporting Eastern countries were in English [28,29]. Lastly, most of the recently published results allowing for causal interpretation, are based on analysis of the individual data [17,19,20,21]. Though offering methodological advantages over aggregated data, which allow for the use of advanced estimation techniques, in particular the ones aiming at appraisal of causality, they have a drawback of not allowing us to analyse impact on the societal level. The above statements have motivated us to conduct a complementary research for Poland. The aim of our work is, therefore, to analyse how SA contributes to SC accumulation in Poland at the regional level, capturing SC dimensions that are not observable on an individual level. This is also interesting in the context of the discussion of whether sport can contribute to SC rather in the bonding form, or also a bridging one [7], with greater potential for having a general, societal scale of impact.

In line with literature cited above, we utilise various measures that reflect particular SC dimensions on an aggregated level, such as: the formation of organisations with community goals (number of foundations and associations, number of Public Benefit Organisations (PBOs) that received the 1% Personal Income Tax (PIT) grants, which all are considered as reflecting both associational behaviour and willingness to work for the benefit of other members of the society in a sustained manner), donations (PIT deductions for donations, reflecting individuals’ propensity to share financial resources with other members of the society), organisation of local community activities (the number of events organised by community centres, clubs and day care centres, which could be responsive to the willingness of members of communities to participate in them and, at same time, would reflect the tendency to institutionalize social life), changes in the organisation of social life at the basic family level (number of marriages and divorces, reflecting dynamics of the social network at its core—the familial social capital, or bonding SC in a very narrow sense [30], participation in public life (turnout in elections, directly reflecting civic engagement), organisation of SAs in sports clubs (number of clubs, reflecting self-triggering development within an associational, institutionalised framework of sports activity) and participation in the sharing economy (number of non-public library readers—persons who are willing to share resources with others [31], implying openness to other member of the society, reflecting creation of a specific cooperative norm).

The list of proposed variables reflects both empirical results mentioned above and theoretical considerations, as discussed by Pawlowski and Schüttoff [7]. In particular, the associational character of sports (especially within sports clubs or team disciplines) is important, in some cases resulting in being exposed to persons from different groups across society, thus contributing to the adoption of cooperative norms, building trust or bridging to further parts of the social networks, which, in turn, could also result in fostered associational behaviour or greater engagement in the sharing economy. Another possible impact takes place through the learning of respect for rules and other persons due to engagement in sports. Due to the limited availability of data, in our research, we took into account the organisation of local community activity, changes in the organisation of social life at the basic family level, participation in public life, and participation in the sharing economy. Although such a SC measure as the number of marriages may be questionable (it refers to the individual rather than the network within the community), it is useful in explaining the circumstances of accumulating SC. After all, wellbeing and the support of one’s nearest and dearest (spouses, partners) can be important in how we interact with others, as well as the kind of relations people create (bonding or bridging).

## 2. Materials and Methods 

This paper analyses the impact of the SA of 19 years old and older adults on accumulating SC in Poland. SA was understood according to the Polish law of 25 June 2010 on sport, Article 2. 1. as “any form of physical activity, which, through ad-hoc or organised participation, contributes to building or improving one’s physical and mental fitness, to developing social relationships and to enhancing sports performance at any level,” and Article 3. 1, “Sports activities shall be conducted, in particular, in sports clubs” [32], and was measured by the number of adults in sports clubs, related to the total population size. In our research, we follow the positive Putnam’s approach to SC, as a mechanism that strengthens the society integration, and bases it on trust, voluntary, relationships and togetherness. The SC was understood as “social networks, shared norms, values and views that facilitate cooperation within and between groups" [33]. and, after Claridge [6], includes dimensions such as trust, norms and principles defining social activities, types of social interaction and dimensions of social networks, group activities, volunteerism, daily interactions with other people, neighbourly relations and sense of community. Therefore, we utilise various measures reflecting the individual dimensions of SC on an aggregated level (establishment of organisations with social objectives, donating money or blood, organisation of community activity, changes in the organisation of social life at a basic family level, participation in public life, organisation of sports activity in sports clubs and participation in the sharing economy). The following hypotheses were made:

1. Similar to the individual level, at the regional one, SA among adults improves SC; however, the relationship between SA and SC at the regional level may not occur for all SC measures. In particular, in line with empirical literature, foremostly, we expect associational behaviour to be positively influenced, which should be reflected in a greater number of associations, foundations or PBO’s receiving 1% tax. Though positive impact on cooperative norms is well documented in the previous research, limited availability of data led us to use of specific measures (e.g., number of non-public library readers, donations deducted from PIT) that can reflect only a part of the possible impact. Therefore, in this case, chances of observing significant results are lower. Moreover, in line with previous results of matching estimation on individuals for Polish data, we do not expect causal relation with turnout in elections.
2. SA performed in sports clubs is a phenomenon of the self-triggering type.

### 2.1. Sources of Data 

A combination of four longitudinal nationwide datasets was used for the analysis. Firstly, the data provided by Statistics Poland (SP) based on the results of research on sports facilities in Poland were used [34]. Data were collected in 2014 using the following forms: KFT-1 (a full population survey on facilities owned by sports clubs), KFT-OB/a (a full population survey on facilities owned by municipalities) and KFT-OB/b (a full population survey on facilities owned by other entities). On their basis we constructed variables that indicate the number of sports facilities within regions in 2014 (including stadiums, fields, sports arenas). By including information about the year when the facilities in the survey were constructed, we also calculated their number in previous years (the survey is conducted every four years). In this way, we constructed a panel variable describing the creation of new sports infrastructure in Polish counties up to and including the year 2014. 

The second data source was the official statistics database Local Data Bank SP (LDB; [35] comprising results of SP surveys and administrative data sources that yielded information concerning the processes subject to analysis, i.e., sports activities undertaken by Poles, including: the number of adults active in sports clubs [36], the number of sports clubs; social capital including: the number of foundations, associations and social organisations registered in the National Court Register (NCR); the number of events organised by centres, community centres, clubs and day care centres; the number of marriages and divorces; turnout in local elections for presidents of cities and mayors; the number of readers of libraries other than public libraries and data used as source of control variables, such as:

- the area of the region, the size of the population and its structure (by age, gender and place of residence);
- sectoral structure of employment (share of agriculture and industry);
- data on income tax revenues Personal Income Tax (PIT) and Corporate Income Tax (CIT);
- the average salary;
- number and structure of entities registered in NCR.

The third source of data was of the administrative character, since it was based on tax declarations collected by the Ministry of Finance, including:

- the list of PBOs [37], which, in particular, the years 2010–2014 received the “1% PIT” grant;
- information on PIT deductions for donations (total values and numbers of taxpayers benefiting from deductions), in particular years (2008–2014) [38]

The fourth source was the National Electoral Commission data on the turnout in the parliamentary elections (from 2007, 2011 and 2015).

Therefore, most of the data used were based either on full-population surveys, including the study on sports clubs and sport infrastructure, or administrative sources with full coverage. An extended description of the data sources is presented in Text S1 (supplement).

We combined this information into one database with a panel structure at the regional level (common classification of territorial units for statistics (NUTS) 4; n=379 counties, corresponding to the total number of counties in Poland in 2006, 2008 and 2010; we have aggregated data from 2012 and 2014, when 380 counties existed, to maintain the consistent structure). In most cases, our analysis covered five time periods (the number of years included in the sample T = 5; i.e., 2006, 2008, 2010, 2012 and 2014). In some cases, we assigned values from the following year (i.e., 2007, 2009, etc.). This concerned variables such as: the number of events organised by centres, community centres, clubs, day care centres and donations (T = 4; 2008, 2010, 2012, 2014), as well as the turnout in parliamentary elections (T = 3; 2010, 2012, 2014) and number of PBOs (T = 3; 2006, 2010, 2014). 

The combined database enabled us to calculate the SA and SC measures described further in this work. All variables are presented in Table 1. 

### 2.2. Statistical Analysis

To investigate and interpret the relationship between SA and SC regionally, we used the instrumental variable (IV) method. 

#### Choice of Methods

We assumed that the complexity of social phenomena such as SC may limit the extent to which factors explaining the variation of the examined phenomenon in time and space can be measured (i.e., the Omitted Variables Bias). The results of the Durbin–Wu–Hausman test confirmed these assumptions. Therefore, the assumptions of the Random Effects model about the lack of correlation of the residuals with the explanatory variables, i.e., the assumption about the exogeneity of explanatory variables, were invalid. Therefore, first of all, we decided to use the Fixed Effects (FE) model. Its application, together with a panel structure, reduced the problem regarding the unobservable heterogeneity of individual regions. Nevertheless, the described problem is only partially corrected by using the FE specification—in a cross-sectional rather than dynamic dimension. Therefore, in addition to the classic ordinary least squares (OLS) method, we used the IV method. [39]. 

### 2.3. Social Capital Measures

We used the following indicators as SC measures:

- establishment of organisations with social objectives (associations per 100 entities registered in the National Court Register, foundations per 100 entities and number of entities receiving 1% PIT from individual taxpayers per 100 entities);
- donating money (or blood) (total PIT deductions resulting from tax deductible donations—in %, in relation to PIT revenues; PIT payers declaring tax deductible donations/100 people);
- number of events organised by local cultural centres, clubs, etc., per 100 people of working age;
- changes in the organisation of social life at a basic, family level (number of divorces and new marriages per 100 people in working age);
- participation in social life (turnout in national parliamentary and local government elections (in %));
- organisation of sports activities in sports clubs (number of sport clubs per 1000 people of working age)—association is one of the dimensions of SC;
- participation in the sharing economy (number of readers of books rented from non-public libraries per 100 people of working age).

### 2.4. Explanatory Variables

In the model we used exogenous explanatory variables such as: population, population density, urban population share, agricultural labour share, manufacturing labour share, share of non-governmental (including companies) entities ratio (in all entities), average salary, revenues from taxes including personal income taxes and corporate income taxes, age structure of the population (under 15, 15–19, 20–24 and 70+ shares) and female to male ratio. Descriptive statistics for this group of variables are presented in Table S1 (supplement).

The endogenous explanatory variable is SA—defined by the number of 19 or more years old adults exercising in sports clubs (related to the size of the working age population) and the instrument variables are based on information on the availability of sports facilities (i.e., the number of sports facilities related to the size of the working age population). 

### 2.5. Implementation of the Modelling Strategy

To implement IV, the two-stage least squares regression (TSLS) was applied, as available in the plm package in the R environment [40]. In line with the assumptions of the method, an equation is estimated in which the dependent variable was the endogenous variable (SA), and the explanatory variables set was comprised of the instrumental and exogenous variables. In the second equation, where the measure of SC was used as the dependent variable, the theoretical values of SA, instead of the observed values of SA, as in OLS, which are calculated using the first equation, were used as the explanatory variable. In other words, SA values taking into account the changes are explained only by exogenous and instrumental variables. 

**Figure 1:**
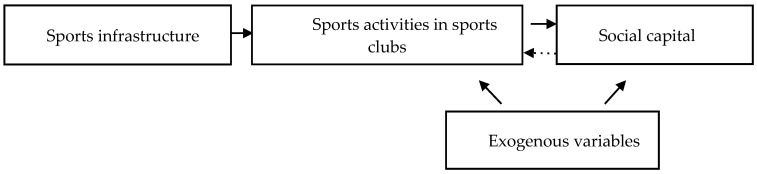
Scheme of modelling the relationship between sports activity in sports clubs and the social capital of Poles, using the instrumental variable method.

As a result, subject to exclusion restriction, appraising causal relations was possible, as shown in Figure 1—i.e., it was possible to explain the changes taking place in SC as a result of the change in SA, resulting from changes in availability of sports infrastructure at the regional level, with the other exogenous variables measuring the local socio-economic development level that was controlled for. 

We chose the instrumental variables from a set of measures describing the number of sports facilities (in line with the subject literature) [7,20]. Based on the value of corrected F statistics proposed by Montiel Olea and Pflueger [41] and using the Stata ivreg2 [42] and weaktest [43] packages, we selected the following for measures: the number of multifunctional fields and the number of football stadiums (both related to the regional working age population). Both instrumental variables were positively correlated with the percentage of people active in sports clubs (also with inclusion of FE for individual counties), which is presented in Table 1. The variance of these variables was ensured by the intense development of sports infrastructure in Poland in the years 2008–2012 within the framework of the My Sports Field: Orliki 2012 programme [44] and by Poland co-organising the Union of European Football Associations (UEFA) European Championship in 2012. In particular, both participation in sports clubs as well as the absolute number and per capita number of facilities of both multifunctional fields and football stadiums grew over the period covered by our analysis.

Our finding that not all types of facilities can constitute a strong instrument is in line with previous findings [20]. At the same time, we argue that exactly these two types of facilities can be assessed as the best in terms of validity. In particular, the multifunctional fields correspond exactly to the type of facilities constructed within the My sports Field: Orliki 2012 programme. The programme was initiated by the central government at the national level and resulted in opening 2604 new facilities between 2008 and 2012. Admittedly, municipalities (local government units, NUTS 5 level) and voivodeships (regional government units, NUTS 2) covered 67% of the expenditure (not including future maintenance costs). Therefore, it can be argued that either interest in sports of the local society or financial considerations have been relevant for the construction of these facilities. We can argue, however, that by utilising longitudinal dataset and FE we control for the unobservable but stable in time social preferences for sports. Furthermore, within the first-stage estimation (as well as the second-stage one), we control for several measures of economic development, including revenue from taxes. Therefore, the changes in resources availability are controlled for. Thirdly, we assess the impact on the county level (NUTS 4), which, apart from big cities, does not correspond to the NUTS 5, or NUTS 2 level. In other words, the policy decisions were often taken at different levels. Lastly, the programme was, in itself, considered one of the flagship projects at the national level, often associated with then Prime Minister of Poland. The propensity to build a field in a given place might have been influenced by political considerations, independent of either affluence or preference for sports at the local level. As for football stadiums (which are separate from football fields), these are often related to professional (or semi-professional) football clubs, even if construction was often financed by municipalities. We argue that, while a ‘trickle-down’ effect here might be possible (i.e., professional sports ‘promoting’ activity at a grassroots level [45]), the decisions to support new facilities is rather driven by state of development of professional sports and adopted policies. While both are dependent on the economic development level, as with multifunctional fields, we made an attempt to control for that.

The results of the overidentification, under identification and endogeneity tests are presented in Table S2 (supplementary). One should bear in mind that the choice of instruments for this was based foremostly on a full five-year time span. As shown in Table S2, the results for variables measured in the shorter range (three or four) should be treated with greater caution due to strength of variables, as well as the IV results for some of the measures (divorces) due to Hansen J statistic value. For the sake of completeness and the fact that for these variables, we find no significant relation; however, we further report all the results.

## 3. Results 

The presented results of the estimation of the IV model allow for the interpretation of the regional level relationships between SC and SA for adult Poles—achieved through exercise in sports clubs. The full results of regression analyses are presented in Table S2 (supplementary).

The results obtained show that SA is positively related to the number of social associations and organisations per capita in the region (Table 2). According to the OLS estimates, with an increase in the number of people exercising (by 1 person in a club per 100 people in the region of working age), the number of social associations and organisations increases by 0.053 (per 100 entities registered in the National Court Register; *p*-value = 0.009). A relationship significantly greater than zero is also maintained in the IV model (the coefficient indicates an increase in the number of associations by 0.911 per 100 entities in the National Court Register; *p*-value = 0.043). 

There is also a positive, although statistically insignificant, link between SA and the increase in the number of entities receiving funds from the 1% PIT donated by taxpayers (in the case of OLS p-value = 0.107). No similar relationship, however, has been shown with regards to the establishment of foundations with social objectives and by donating money or blood, both in terms of the number of individuals benefiting from tax deductions on this account and the sum of deductions. SA was not connected to the increased organisation of local community activities either (measured by the number of events organised by centres, houses, cultural centres, clubs and day care centres). 

According to the IV estimation, an increase of 1 person performing physical exercise per 100 people of working age means 0.326 fewer marriages (for the same population; *p*-value = 0.039). 

The relationship between SA and the participation of Poles in public life is ambiguous. While the turnout in local government elections is positively correlated with SA (1/100 more people exercising means a higher turnout by 0.361 percentage points; *p*-value = 0.077); in parliamentary elections (at a national level) such a relationship does not appear. 

On the other hand, SA has a minor positive impact on the number of users of non-public libraries. The IV estimation indicates that an increase in the number of people of working age performing physical exercise by one in 100 results in an increase in the number of readers (by 7.293; *p*-value = 0.047). 

The formation of sports clubs seems to be strongly responsive to the Polish demand for SA. According to the OLS estimate, one additional person exercising in a sports club (per 100 people of working age in the regional population) means an increase in the number of clubs by 0.236 (per 1000 people of working age; *p*-value < 0.001). According to the IV estimate, the number of clubs increases by 0.450 (per 1000 people of working age; *p*-value = 0.005). 

## 4. Discussion

Despite the theoretical support of the literature on the relationship between SA and health [46] and community building [47], still not much is known about its role in the accumulation of SC. According to US reports, the role of sport in this respect is ambiguous and needs to be verified [48]. Although the tradition of such thinking is still strong [49], it is present in numerous political statements [50] and is implied in much of the scientific work conducted on sport and SC [51]. This study can be considered as another one that contributes significantly to the broadening empirical evidence on the subject matter by ascertaining whether, at the regional level in the adult population of Poles, SA (understood as exercises in sports clubs) play a part in the accumulation of SC. 

Our results confirm the hypothesis about SA contribution in SC accumulation. This supports Uslaner’s [52] earlier belief that sport can be a suitable arena for SC strengthening. It also supports Schulenkorf’s [53] evidence that sports projects and events can contribute to inter-community development and increase SC resources. Finally, it is consistent with the results of Fu et al. [54] and Mummery [55] depicting physically active people as people with higher SC. 

Of course, the influence we found is quite remote. Sport as a type of physical activity (in itself) does not accumulate SC [20]—this is determined by the social and organisational context, e.g., practising sport with friends, neighbours and families [49]. Exercising with them, and sharing hardships and circumstances increases the level of trust and improves social relations—essential for the functioning of society and crucial for SC. According to Putnam [2], relationships and trust built up by participation in sport facilitate the creation of new social networks and the maintenance of existing ones. According to Perks [56], they also foster the involvement in community activities outside sport. Our results show that changes in SA (increase in the number of people exercising of 1/100 people of working age in the region) are associated with an increase in the value of SC indices such as: number of social associations and organisations per capita in the region (statistically significant for OLS and IV), and the use of non-public libraries (statistically significant for IV).

This leads us to positively verify the first hypothesis and to conclude that at the regional level, SA in adults improves SC indicators. However, we would like to draw attention to the fact that over the study period, we only recorded for some indicators/SC measures statistically significant changes or changes signifying trends. In line with the caveat to the first hypothesis, we have observed that, according to the literature [57], SC is a phenomenon characterised by a high level of inertness. Therefore, the relationship observed only exists with some of the SC measures, i.e., the changes most sensitive to SA that can be seen during the investigation period. However, this draws attention to the need for future estimation of the changes in SC in a long-term perspective. Perhaps then the effect achieved will be strong enough to be recorded for more SC indicators. 

As expected, the effect on the associational behaviour is observed, but the relationship of SA with the formation of organisations with social objectives is not so clear. While we observe that the increase in the number of people engaged in exercising (by 1/100 people) has a positive impact on the increase in the number of social associations and organisations (by 0.053 per 100 entities registered in the National Court Register), we do not find such a relation in the case of establishing foundations. Perhaps the point is that foundations in Poland are not as widespread as associations (in 2018, 26,000 foundations were registered vs. 117,000 associations; [58]. Perhaps this is a consequence of the conviction that the foundation functioning model does not require the involvement and participation of anyone else but the Board (it is enough to support it financially). Or maybe this is due to the fact that foundations have more diversified goals, not only pro-social—i.e., they can, for example, conduct business activity in order to raise funds for their goals. 

According to the Schüttoff et al [17], regular SA is accompanied by a greater tendency to belong to civic organisations, local governments and political parties. Delaney and Keaney [12] point to the small, albeit statistically significant, correlation of sports club membership with political commitment, while Seippel [59] remarks on overall political interest and voting. For Poland, however, no significant impact of SA on propensity to vote in elections was found [21]. In our study, the increase in the number of people exercising in sports clubs (by 1/100 people) is only associated with a higher turnout in local government elections (by 0.361 percentage points). However, we do not find any connection with turnout in parliamentary elections, nor is the relation confirmed by the IV estimation. This can be explained, according to Seippel [59], by the fact that the SC arising from participation in sports organisations is more often associated with general social obligations (e.g., generalised trust) than with politicised issues (e.g., trustworthiness of politicians or satisfaction with democracy). At the same time, local government elections can be seen as less “politicised” than elections at the central level, which may explain the lack of significant correlation between turnout in the national election and SA in our research. It is also possible that this is the result of the inertia mentioned above, e.g., related to the Poles’ distrust, which is connected with tradition and has a cultural dimension (still existing in new conditions that would justify regaining trust [60]. Surveys conducted by the Centre for Social Opinion Research [61] show that Poles are extremely distrustful—they have little confidence in state institutions, political parties or politicians. This fosters political passivity among citizens (in 2006, as much as 42% declared themselves indifference regarding how power is exercised and whether the government is democratic or undemocratic [62]. This is confirmed by the European Social Survey [12,63], showing that among all European countries, Poland has the lowest level of generalised trust and civic involvement. However, the involvement in SA, especially limited to sport clubs, is too weak to reduce this barrier, even more so in the relatively short period of time. It should also be mentioned that declining confidence is a trend (and also a problem) that can be seen throughout Europe. As shown by Pattie et al. [64], the participation of citizens in conventional political activities such as voting, contact with politicians and participation in political meetings has clearly decreased in recent years. All this shows that participation in political activities remains a relatively minor aspect of social life in Poland. 

In line with expectations, the results regarding measures related to cooperative norms are mixed. The analysis shows that an increase in the number of people pursuing physical exercise in the region by one in 100 people of working age results in an increase in the number of readers by 7.293 (although the OLS estimate suggests a weaker effect, which suggests caution while interpreting the IV results). This fact may have further consequences. According to Johnson [65], social interactions taking place in libraries (e.g., building patrons’ trust in the library and its staff, connecting people to both community and library resources, providing social support for patrons, reducing social isolation, helping patrons gain skills in order to function in an increasingly online world and providing a positive place for neighbourhood residents to gather) support the accumulation of SC. However, the role of libraries in accumulating SC can increase, and SC itself can be important for the functioning of libraries [66]. 

The results of the IV estimation imply that a higher percentage of Poles active in sports is associated with a lower (by 0.326) number of marriages. However, it should be borne in mind that the OLS estimates for either the number of marriages or divorces did not differ significantly from zero. With that caveat in mind, our results indirectly point to the growing phenomenon of preference, among physically active people, for being single. This may testify to contemporary changes in people’s lifestyles, i.e., a conscious choice of an alternative way of life, which—as Żurek [67] proposes —is accompanied by: the concept of a life goal, a network of friends and an increase in volunteerism. Singles in Poland have many and various friends (18 on average) and as much as 17.3% are members of some social organisation [67]. 

Moving on to the discussion of the second hypothesis, that SA pursued in sports clubs is a particular phenomenon of the self-triggering type, we find its positive verification.

In our research, an increase in the number of people exercising (1/100 in the region) means an increase in the number of sports clubs by 0.236 (per 1000 inhabitants of the region at working age, in the case of OLS estimation) or by 0.450 (in the case of IV estimation). This shows their strong responsiveness to the demand for SA. The research of Eime, Young et al. showed that participation in solitary as well as team sport can enhance adults’ mental health, which contributes to their personal growth and is an important factor for the social health, development and engagement [24]. In our opinion, sport through its numerous functions (educational, recreational, integrative, promotional) and community character shapes people. It unlocks personal potential of people, teaches them cooperation, healthy competition and general life activity. This activity means that an individual can actively regulate one’s relations with the environment—not only adapt to it, but also cause changes in it. Of course, the direction and intensity of this activity is determined each time by the individual’s situation (internal state and the state of the environment). According to the theory of adaptation (to old age), activity works on the principle of feedback—it is an expression of a positive response to life tasks, and at the same time, by strengthening self-esteem, it becomes a driving force for further development. 

The beneficial impact of engagement in sports activity, either through stimulating effect on personal activeness or the extension of social networks and greater openness to needs of other members of society, can lead to a greater propensity to create new organisations in a given community. Therefore, also with the conclusions of previously cited publications taken into account, our study confirms that the relationship between social capital and sports activity is in fact reciprocal, and that sport, therefore, might be an asset of greater value for society that what would result only from the health considerations. 

### Strengths and Limitations

The main advantage of this study is that, according to our knowledge, for the first time in Poland (a post-transformation country) we conducted an analysis of the impact of adult SA on the SC accumulation on aggregated level. This is a context of research that differs significantly from that of Western European and North American countries or developing countries—both in terms of SA (especially the availability of infrastructure and changes to it in time) and SC. For this purpose, we have used all available, according to our knowledge, nationwide databases: official statistics of Statistics Poland and tax data of the Ministry of Finance and election data of the National Electoral Commission. The analysis was carried out regionally (NUTS 4), which made it possible to capture the SC dimensions not observed at an individual level. The panel data structure made it possible to calculate the SA and SC measures. We are fully aware that the use of only selected SC indicators may lead to a distortion of the actual picture and omission of many important social interactions. Unfortunately, the limited availability of data at the regional level (guaranteeing an appropriate number of observations for econometric analyses) did not facilitate a broader view—both in this respect and in the analysis of a more diverse group of people, i.e., those undertaking physical activity outside sports clubs. For future studies, we see a need to take such people into account, as well as to consider the indicators depicting different aspects and types of SC. Furthermore, also the limited data availability did not allow for estimation of long-term effects in the present study. The case analysed here concerned Poland and, due to all the country-specific circumstances regarding both participation in sports clubs and particular measures of social capital, the results of this study should be interpreted with caution, especially in terms of generalisation of the implications.

## 5. Conclusions

The results of this study prove that we can positively answer the question posed in the title of the paper and state that, at the regional level, adults’ SA improves SC. Sports clubs and the well-chosen use of specific types of sports infrastructure can promote the accumulation of social capital and further organisation of this sector. Therefore, specific actions supporting SA in sports clubs might be conducive to the SC accumulation. Not all changes in SC measures are observable in the same time perspective. That is why measures and indicators of social capital should be widely chosen, due to the inertia of the phenomenon subject to research, and it is also important to estimate the changes of SC in a long-term perspective in the future. The use of knowledge on the self-triggering character of SA can be a valuable tool for social impact both regionally and beyond, strengthening the interpersonal social bonds and giving rise to the social development.

## Figures and Tables

**Table 1:** Descriptive statistics for dependent, endogenous and instrumental variables.

Variable	T	Mean	St. Dev.	Min	Max
Sports activity					
Number of people training at sports clubs/100 people	5	1.07	0.58	0.00	5.50
Sports infrastructure					
Multifunctional fields/10,000 people	5	0.83	1.05	0.00	7.11
Football stadiums/10,000 people	5	0.61	0.69	0.00	7.25
Social capital					
Establishment of organisations with social objectives					
Associations/100 entities	5	2.89	1.01	0.74	7.35
Foundations/100 entities	5	0.17	0.14	0.00	1.45
Number of entities receiving 1% personal income tax from individual taxpayers/100 entities	3	0.14	0.11	0.00	1.10
Donating money (or blood)					
Total Personal Income Tax deductions resulting from tax deductible donations (in %; in relation to PIT revenues)	4	1.00	0.56	0.20	4.39
Personal Income Tax (PIT) payers declaring tax deductible donations/100 people	4	1.09	0.67	0.20	5.96
Organisation of local community activities					
Number of events organised by local cultural centres, clubs, etc./100 people	5	0.93	0.67	0.00	7.24
Changes in the organisation of social life at a basic level					
Number of divorces/100 people	5	0.25	0.08	0.05	0.63
Number of new marriages/100 people	5	0.92	0.14	0.64	1.42
Participation in public life					
Turnout in national parliamentary elections (in %)	3	47.44	6.64	33.40	88.27
Turnout in local government elections (in %)	3	48.50	5.67	27.58	65.86
Organisation of sports activities in sports clubs					
Number of sports clubs/1000 people	5	0.66	0.32	0.11	3.05
Participation in the sharing economy					
Number of readers of books rent from non-public libraries/100 people	5	4.49	9.47	0.00	144.50

Note: T is the number of years included in the sample (for T = 5—2006, 2008, 2010, 2012 and 2014 are included; for T = 4—2008, 2010, 2012 or 2014; for T = 3—2010, 2012 and 2014 or 2006, 2010 and 2014). All per capita categories are calculated with respect to working age population size. All entities are based on the number of registered entities.

**Table 2:** Estimates of regression coefficient for the number of people over 18 years of age exercising in sports clubs in a region (per 100 people of working age)— Ordinary Least Squares (OLS) and Instrumental Variables (IV).

	Variable	OLS Coefficient	*p* Value for OLS	IV Coefficient	*p* Value for IV	OLS R^2^	IV R2
Establishment of organisations with social objectives							
Foundations per 100 entities in the National Court Register	Foundations/100 entities	−0.004	0.416	−0.055	0.359	0.607	0.563
Social associations and organisations per 100 entities in the National Court Register	Associations/100 entities	0.053 **	0.009	0.911 *	0.043	0.742	0.456
PBOs receiving 1% PIT, per 100 entities in the NCR	Number of entities receiving 1% personal income tax from individual taxpayers/100 people	0.008	0.107	0.128	0.520	0.444	0.182
Donating money (or blood)							
Total PIT deductions on donations (in %; in relation to PIT income)	Total Personal Income Tax deductions resulting from tax deductible donations (in %; in relation to PIT revenues)	0.037	0.272	−0.660	0.183	0.079	0.004
PIT payers deducting donations per 100 people of working age	Personal Income Tax payers declaring tax deductible donations/100 people	0.012	0.421	0.244	0.256	0.596	0.514
Organisation of local community activities							
Number of events organised by centres, houses, community centres, clubs and day-care centres; per 100 persons (year later)	Number of events organised by local cultural centres, clubs, etc./100 people	−0.026	0.537	−0.091	0.847	0.015	0.013
Changes in the organisation of social life at a basic level							
Divorces per 100 people	Number of divorces/100 people	0.004	0.362	0.037	0.479	0.164	0.129
Marriages per 100 people	Number of new marriages/100 people	0.006	0.476	−0.376*	0.039	0.587	0.256
Participation in public life							
Turnout in parliamentary elections (in percentage points; one year later)	Turnout in national parliamentary elections (in %)	0.232	0.333	5.092	0.115	0.457	0.318
Turnout in the elections for presidents of cities/mayors (in percentage points)	Turnout in local government elections (in %)	0.361	0.077	1.294	0.622	0.241	0.245
Organisation of sports activities in sports clubs							
Number of sports clubs per 1000 inhabitants	Number of sports clubs/1000 people	0.236 ***	<0.001	0.450 **	0.005	0.383	0.368
Participation in the sharing economy							
Number of readers of libraries other than public libraries per 100 inhabitants (year later)	Number of readers of books rent from non-public libraries/100 people	0.108	0.825	7.293 *	0.047	0.213	0.098

Note: PBO – Public Benefit Organizations, PIT - Personal Income Tax, NCR – National Court Register. The exercise rate and all variables per capita are related to the working age population. Significance: *** <0.001, ** <0.01, * <0.05, <0.1. Robust standard errors are reported (Arellano method, type HC1).

## Supplementary Materials

The following are available online at https://www.mdpi.com/1660-4601/17/14/5257/s1, Text S1: Extended description of data used in the study, Table S1: Descriptive statistics for exogenous variables, Table S2: Full regression results, File S1: Dataset.

## Abbreviations

SC	social capital
SA	Sports Activity
NUTS 4	Nomenclature of Statistical Territorial Units
SP	Statistics Poland
KFT-1	survey on facilities owned by sports clubs
KFT-OB/a	survey on facilities owned by municipalities
KFT-OB/b	survey on facilities owned by other entities
LDB	Local Data Bank
NCR	National Court Register
PIT	Personal Income Tax
CIT	Corporate Income Tax
PBOs	Public Benefit Organisations
T	time periods
IV	instrumental variable
FE	Fixed Effects
OLS	ordinary least squares method
TSLS	two-stage least squares regression
NGOs	nongovernmental organisations
